# Single-Step Metal-Free Grafting of Cationic Polymer Brushes on Fluorescent Nanodiamonds

**DOI:** 10.3390/ma11081479

**Published:** 2018-08-20

**Authors:** Shingo Sotoma, Feng-Jen Hsieh, Huan-Cheng Chang

**Affiliations:** 1 Institute of Atomic and Molecular Sciences, Academia Sinica, Taipei 106, Taiwan; fengjen.hsieh@gmail.com; 2 Department of Chemical Engineering, National Taiwan University of Science and Technology, Taipei 106, Taiwan

**Keywords:** cationic polymer, cell labeling, fluorescence imaging, nanodiamond, ring-opening reaction

## Abstract

Cationic polymers are often employed in conjugation with nanomaterials, and the resultant hybrids are useful for various bioapplications. Here, a single-step metal-free method for the synthesis of fluorescent nanodiamonds (FNDs) conjugated with cationic polymer brushes is reported. Distinct from the common methods such as atom transfer radical polymerization and reversible addition fragmentation chain transfer, our ring-opening-polymerization-based method is simple and less time consuming and hazardous. Infrared spectroscopy, thermogravimetric analysis, zeta potential, and dynamic light scattering confirmed the synthesis. The produced FND-polymer brushes showed markedly higher cell labeling and internalization efficiency without noticeable cytotoxicity. Our method is general and applicable to other nanoparticles as well for uses in diverse research areas.

## 1. Introduction

Surface modification with cationic polymer brushes is one of the key technologies to control the properties of nanoparticles. To date, a variety of polymer brushes have been developed and used for diverse bioapplications [1,2]. Among them, nanoparticles with cationic polymer brushes have been utilized as antibacterial agents [3] as well as for efficient cell uptake [4] and drug/gene delivery into cells [5], exploiting their positive surface charge [1]. In order to graft cationic polymers on the surface of nanoparticles, atom transfer radical polymerization (ATRP) [6] and reversible addition fragmentation chain transfer (RAFT) polymerization [7] are often used. However, these methods typically involve several steps of organic synthesis and are time-consuming. Moreover, the methods often require transition metal catalysts such as Cu^+^ ions for the polymerization. Since these transition metal ions are toxic to living organisms, it is better to avoid them in the synthesis. To our knowledge, the development of a facile, ideally single-step, and metal-free method for producing nanoparticles with cationic polymer brushes has not been reported so far, but is critically important for applications in the life sciences.

To address this challenge, ring-opening polymerization (ROP) offers a solution [8]. It has been reported that glycidol can be polymerized on the surface of nanoparticles and forms branched polymer brushes without the need of metal catalysts [9,10,11,12]. The mechanism of ROP is the consecutive nucleophilic reaction between an OH group and a carbon atom in the epoxy group. Based on this finding, we propose a novel synthetic route to produce nanoparticles surface-functionalized with cationic polymer brushes by simply reacting the particles which have nucleophilic groups and glycidyltrimethylammonium chloride (GTMA) that contains an epoxy ring and a cationic trimetylammonium group. The synthesis can be finished in a single-step, requiring no metal catalysts, and thus the synthesized nanoparticles are non-toxic to cells and organisms.

The nanoparticle platform used to demonstrate this method is the fluorescent nanodiamond (FND). FNDs are carbon-based nanoparticles, having stable fluorescence, low toxicity, and physicochemical inertness [13,14,15]. Taking advantage of these properties, single particle tracking in cells and long-term cell tracking in animals have been performed [16,17,18]. For such applications, efficient labeling of the cells by internalization of FNDs is important. However, the efficiency is typically moderate because both acid-treated FNDs and the cell surface are negatively charged. Hence, if the surface of FNDs can be covered with cationic polymer brushes, highly efficient labeling of the cells with FNDs can be achieved. Since the nucleophilic groups (e.g., COOH) are formed on the surface after acid treatment [19], FNDs are optimal for this ring-opening-polymerization-based cationic polymer grafting. Distinct from protein or lipid coatings [20], the coatings with polymer brushes are generally stable because the coating structure is maintained by covalent bonding. Here, we report a single-step metal-free method for the synthesis of cationic polymer brush coated FNDs and demonstrate their high cell labeling and internalization efficiency.

## 2. Materials and Methods

### 2.1. Preparation of FND

Nanodiamond powders (~100 nm, Element Six, London, UK) containing ~100 ppm of atomically isolated nitrogen were irradiated by He^+^ ion (40-keV), annealed at 800 °C for 2 h under reduced pressure, and air-oxidized at 450 °C for 2 h, yielding FNDs. Further details are described elsewhere [21]. To eliminate surface contamination, FNDs were treated with a mixture of H_2_SO_4_:HNO_3_ (3:1 v/v) at 70 °C for 3 days and then washed with deionized distilled water (DDW) three times. Next, the solution of FND slurry was replaced with 0.1 M NaOH and stirred for 1 h at 90 °C. After centrifugation and wash process, the solution of FND slurry was replaced with 0.1 M HCl and stirred for 1 h at 90 °C. Finally, the FNDs were centrifuged and washed three times with DDW, yielding bare FND (FND-bare).

### 2.2. Synthesis of FND-PEGTMA, FND-HPGTMA, and FND-PEI

FNDs (5 mg) were dispersed in glycidyltrimethylammonium chloride (GTMA) or the mixture solution of GTMA and glycidol (Sigma-Aldrich, St. Louis, MO, USA) at a different volume ratio of 1.5/0.5, 1.0/1.0, and 0.5/1.5 (mL/mL). Note that moisture in GTMA and glycidol was removed by rotary evaporation before use. The mixtures were then sonicated for 5 min, and stirred for 24 h at 120 °C under nitrogen atmosphere. After the reaction, the resulting gel was diluted with DDW, and centrifuged at 15,000 rpm for 5 min to collect FND-PEGTMA or FND-HPGTMA. Then they were washed four times to remove by-products and unreacted materials.

FNDs (1 mg) and Polyethylenimine (PEI, Mn ~1200, average Mw ~1300, Sigma-Aldrich, 1 mg) were dissolved in DDW (1 mL), sonicated, and gently mixed for 1 h. Unbound polymers were removed from the FND-PEI conjugates by repeated centrifugation/wash processes with DDW.

### 2.3. Characterization of FND Samples

Fourier transform infrared spectroscopy (FTIR, Bruker, Karlsruhe, Germany) spectra were obtained on a Bruker VERTEX 70 using standard KBr-pellets in the range of 2200–400 cm^−1^ with a resolution of 4 cm^−1^. Thermogravimetric analysis (TGA) measurements were carried out in air at a heating rate of 10 °C min^−1^ using a Thermal Analyzer (TA Instruments, New Castle, DE, USA). The average sizes and zeta potentials of FNDs were measured using a Delsa Nano C (BECKMAN COULTER Inc, Brea, CA, USA). Size distributions are represented by the particle number.

### 2.4. Cell Culture, Labeling, Magnetically Modulated Fluorescence (MMF), and Confocal Fluorescence Microscopic Observation

HeLa cells were cultured in Dulbecco’s modified Eagle’s medium (DMEM) with 10% fetal bovine serum and 1% penicillin at 37 °C in 5% CO_2_ incubator. For magnetically modulated fluorescence analysis, HeLa cells were plated at a density of 5 × 10^5^ cells per 35 mm dish in DMEM and cultured for 22–26 h in the incubator. After incubation, the culture medium was subsequently replaced with an FND-containing medium (10, 20, 50, or 100 μg/mL). Note that the concentrations of FNDs were determined in terms of the fluorescence intensity, namely, polymer coating was not taken into account. After incubation for 1 h, cells were washed with PBS extensively and further incubated for three hours. The cells were then trypsinized, collected, counted, and suspended in DDW. After sonication, the fluorescence intensity of FNDs were measured and analyzed by home-built MMF spectrometer [18]. Briefly, fluorescence from FNDs can be modulated by applying a time-varying external magnetic field frequency to minimize background autofluorescence. We applied a magnetic field of 20 mT at a modulation frequency of 10 Hz, and then applied a fast Fourier Transform (FFT, Hitachi Ltd., Tokyo, Japan) to extract concentration information from the measured fluorescence intensities. The technique enables highly sensitive detection of FNDs and allows us to determine the mass of FNDs, and subsequently the absolute number of FNDs in solution can be estimated. For confocal microscopic observation, HeLa cells were plated at a density of 2 × 10^5^ cells per 35 mm dish, treated with FND samples, and stained with Hoechst 33342. Fluorescence images were observed with a Leica SP8 (Leica Camera AG, Wetzlar, Germany) equipped with an oil-immersion objective (63×, NA 1.4, Leica Camera AG, Wetzlar, Germany), a white light laser (Hoechst 33342, EX:405 nm; FND, EX: 532 nm), a photomultiplier (Hoechst 33342, EM: 415–485 nm) and hybrid detector (FNDs, EM 600–750 nm). Logical sizes and pixel sizes of X and Y dimensions were 512 × 512 and 0.361 μm × 0.361 μm, respectively, and voxel size of Z dimension is 0.5 μm.

### 2.5. Cell Viability Assay

Dehydrogenase activity detection in viable cells was measured with the colorimetric assay cell counting kit-8 (CCK-8, Dojindo, Tokyo, Japan). HeLa or MCF7 cells (5000 cells in 100 μL culture medium) were seeded in 96-well plates for 22–26 h. FND samples were then added to cells and incubated for 22–26 h at 37 °C. The absorbance was measured at 450 nm by using a plate reader (Multiskan, Thermo, MA, USA) one hour after the addition of CCK-8.

## 3. Results and Discussion

**Scheme 1:**
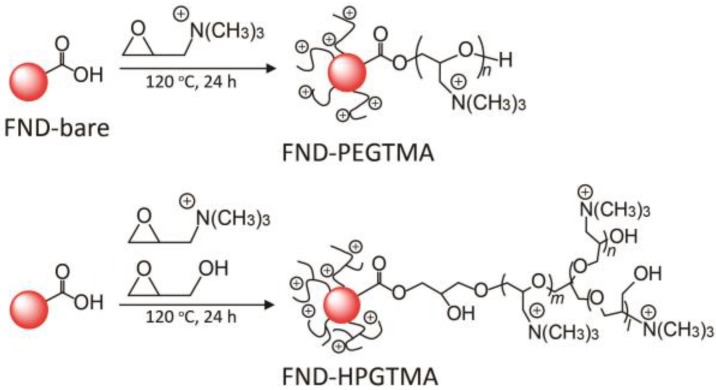
Synthetic of polyethylene glycol with trimethylammonium on fluorescent nanodiamond (FND-PEGTMA) and hyperbranched polyglycerol with trimethylammonium on fluorescent nanodiamond (FND-HPGTMA).

Scheme 1 shows the synthetic route of two types of FNDs with cationic polymer brushes; (1) polyethylene glycol with trimethylammonium on FND (FND-PEGTMA) and (2) hyperbranched polyglycerol with trimethylammonium on FND (FND-HPGTMA). FND-PEGTMA and FND-HPGTMA have linear and branched polymer chains, respectively. In FND-HPGTMA, glycidol contributes to introducing a branch structure in the polymer. FND-PEGTMA was synthesized by dispersing FNDs in GTMA and the solution was heated to 120 °C for 24 h under a nitrogen atmosphere. As for the synthesis of FND-HPGTMA, the reaction conditions were the same as that of FND-PEGTMA except that glycidol was added into the reaction solution. Several concentrations of glycidol (GTMA/glycidol = 75, 50, and 25 vol %) were tested to optimize the synthesis. Further experimental details can be found in the Experimental Section.

**Figure 1:**
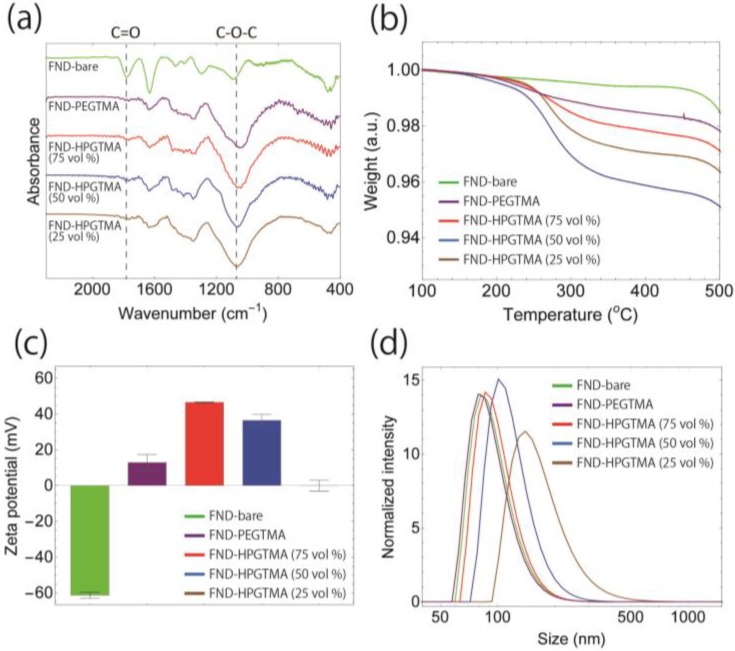
Characterization of fluorescent nanodiamond (FND) samples with (**a**) Fourier transform infrared spectroscopy (FTIR); (**b**) thermogravimetry (TG); (**c**) zeta potential analysis; and (**d**) dynamic light scattering in DDW. Data of FND-bare, FND-PEGTMA, and FND-HPGTMA (GTMA/glycidol = 75, 50, and 25 vol %) are represented in green, purple, red, blue and brown, respectively. Values in (**c**) are means ± standard deviations of three measurements.

Results of the synthesis were examined by Fourier transform infrared spectroscopy (FTIR), thermogravimetric analysis (TGA), zeta potential, and dynamic light scattering (DLS). After the reaction, the absorption intensity of the –C–O–C– stretching modes of the polymer around 1100 cm^−1^ in the FTIR spectra were found to increase for both FND-PEGTMA and FND-HPGTMA, compared to that of bare FND (FND-bare) as shown in Figure 1a. In contrast, the relative intensity of the –C=O stretching of the surface –COOH groups was diminished after polymer grafting. In order to determine the amount of grafted polymers, we conducted TGA (Figure 1b). The polymers started to decompose at ~250 °C and disappeared nearly completely at 400 °C, showing a weight percentage of roughly 1–4% for GTMA/glycidol = 75, 50, and 25 vol %.

We next investigated the zeta potentials of the samples (Figure 1c). Acid-treated FNDs have a strong negative potential of around −60 mV owing to the presence of −COOH groups on the surface. For FND-PEGTMA, the potential was changed to the positive as +13 mV. In comparison, the potential was significantly increased to +47 mV for FND-HPGTMA (75%). The reason for this increasing potential might be as follows: The shape of the polymer chain of FND-PEGTMA is linear and the chain might not be long enough to cover the whole surface area. On the other hand, FND-HPGTMA has a 3D branch structure, enabling it to cover the large surface area, which gives a higher number of cationic groups on the surface resulting higher cationic potential to the particles. However, further addition of glycidol, FND-HPGTMA (50% and 25%), induced the decrease of positive charge because of the reduced amount of the original cationic charge. Figure 1d shows the particle size distributions of these five FND samples in water. The hydrodynamic sizes are increased with the increasing ratio of glycidol/trimethylammonium used for FND-HPGTMA preparation. The large distribution of FND-HPGTMA (25%) in size reflects the formation of aggregation (Figure S1), which might be due to their low zeta potential (Figure 1c).

Collectively, we have successfully developed a single-step, metal-free method for the synthesis of FNDs with cationic polymer brushes. Under the experimental conditions described herein, the GTMA/glycidol ratio of 75% gave the highest positive zeta potential value and these conjugates, indicated as FND-HPGTMA, are used in subsequent experiments. Stability of FND-HPGTMA under various ion concentration and pH condition was studied (Figure S2).

**Figure 2:**
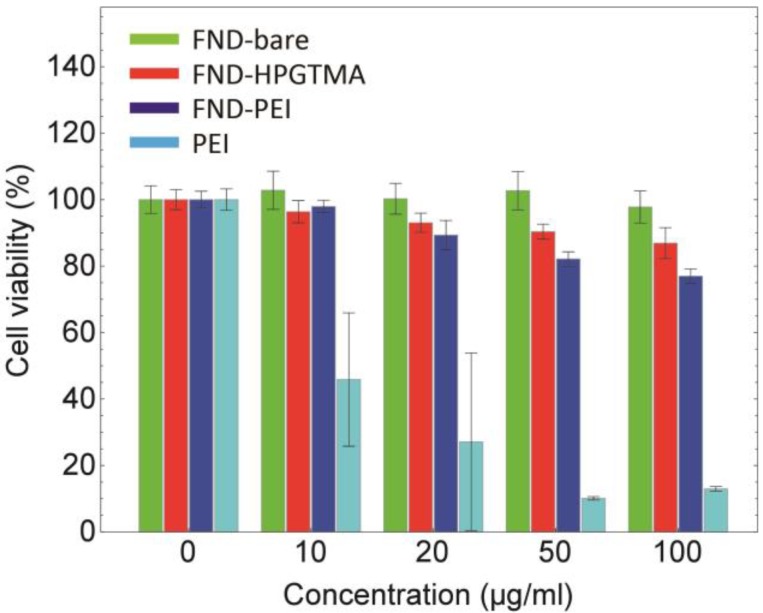
Cell viability testing of HeLa cells treated with FND-bare, FND-HPGTMA, and FND-PEI, and PEI for 24 h using CCK-8. Values are means ± standard deviations of three measurements. PEI: Polyethylenimine.

We performed the CCK-8 assay to test the cytotoxicity of FND-HPGTMA (Figure 2) with HeLa cells. FND-bare, polyethyleneimine (PEI, a commonly used cationic polymer for conjugation), and PEI-coated FND (FND-PEI) were used as controls. Details of FND-PEI preparation are given in experimental section and Figure S3. Note that the concentration of FNDs was calculated in terms of core diamond particles without considering the surface-modified groups. No observable cytotoxicity was found for the FND-bare, whereas PEI is highly toxic to the cells. As for FND-HPGTMA, in spite of its strong cationic property, 87% of the cells were still alive even after the high concentration treatment at 100 μg/mL. Compared to the cell viability of 77% for FND-PEI at the same particle concentration, the cytotoxicity of FND-HPGTMA is much lower. The moderate cytotoxicity of FND-HPGTMA was further confirmed with MCF7 cells, a breast cancer cell line (Figure S4).

**Figure 3:**
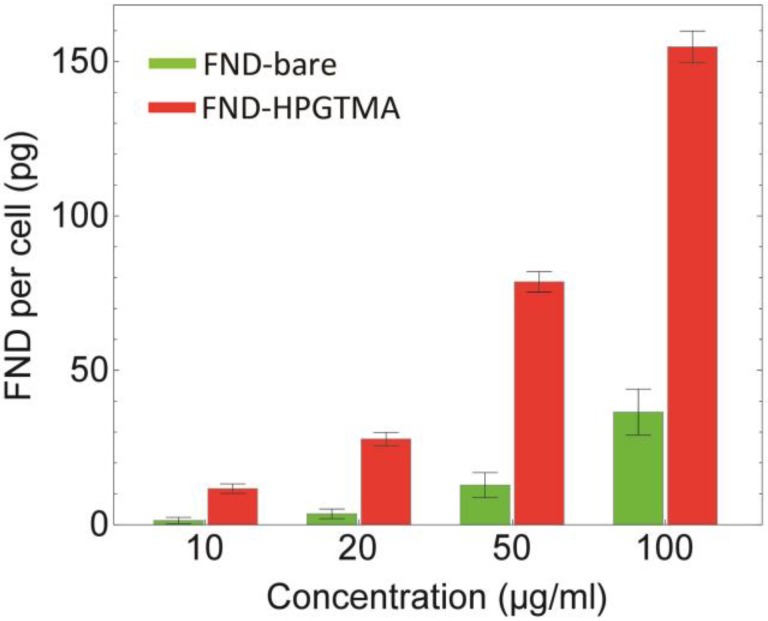
Concentration-dependent labeling of HeLa cells with FND samples analyzed by magnetically modulated fluorescence. Values are means ± standard deviations of three measurements.

Next, we demonstrated a markedly high cell labeling efficiency of FND-HPGTMA. For obtaining a quantitative measure, we performed magnetic modulation to realize background-free fluorescence detection of FNDs [18,22]. Details of the magnetic modulation method are found in the Materials and Methods section. Distinct from flow cytometric analysis, the technique allows us to determine the absolute number of FNDs within the cells [18]. To conduct the experiments, HeLa cells were incubated with FNDs at concentrations of 10–100 μg/mL for 1 h, followed by an extensive wash with phosphate-buffered saline (PBS). The number of FNDs internalized by HeLa cells and anchored on cells membrane was then analyzed by fluorescence intensity measurement (Figure 3). We observed that the cells treated with FND-HPGTMA possessed markedly more FNDs (ca. 7-fold), compared to the cells treated with FND-bare. A single cell treated with 100 μg/mL of FND-HPGTMA contains roughly 8 × 10^4^ FND particles, assuming that the particles are spherical and uniform in size. In comparison with the cell labeling with protein-coated FNDs [18], FND-HPGTMA can realize highly efficient cell labeling.

**Figure 4:**
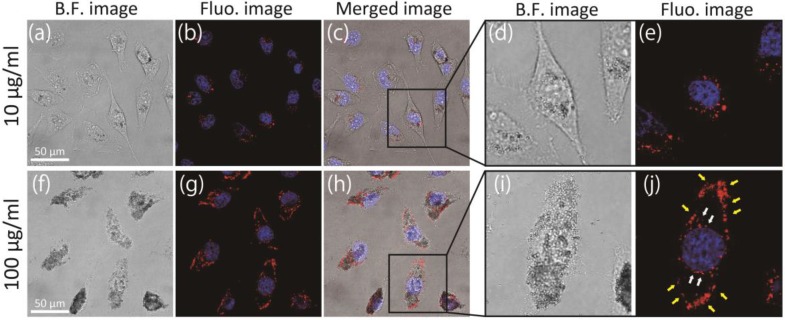
(**a**–**j**) Bright-field, confocal fluorescence (FNDs in red and nuclei in blue) on the level of cell nuclei, and merged images of HeLa cells treated with FND-HPGTMA at 10 μg/mL (**a**–**e**) and 100 μg/mL (**f**–**j**). Close-up images of black square areas in (c,h) are also shown (d,e,i,j). White and yellow arrows in (j) indicate internalized and membrane-anchored FNDs, respectively.

Finally, using confocal fluorescence microscopy, we investigated whether FND-HPGTMA is taken up by cells or anchored on the cell membrane for the treatment at two different concentrations (Figure 4). For the cells treated with 10 μg/mL at 37 °C for 1 h, most of the FNDs were internalized inside cells and localized around nuclei (Figure 4a–e). Few FNDs were observable along with the cell membrane. Confocal images from different focal planes are shown in Figure S5, providing clear evidence for the internalization of FND-HPGTMA into the cells. On the other hand, the cells treated with 100 μg/mL under the same condition, FNDs stayed not only inside the cells but also on the cell membrane, which are indicated by white and yellow arrows, respectively (Figure 4f–j). The result seems to imply that this condition is beyond the limit of the cell internalization system. These membrane-anchored FNDs might disturb cell-cell interactions and, hence, one needs to be cautious when assessing the labeling condition in practical applications. In Figure S6, we represented confocal microscopic images of HeLa cells treated with FND-bare and FND-HPG at 100 μg/mL as a comparison.

## 4. Conclusions

We have successfully developed a single-step metal-free method for facile production of FNDs with cationic polymer brushes. Compared to ATRP or RAFT, our method is simple and less time-consuming and hazardous. Since the method involves only ring-opening polymerization, it is widely applicable to other nanoparticles as well [10,23,24,25,26,27]. Specifically, FND-HPGTMA (FND with polymer brushes consisting of hyperbranched polyglycerol with trimethylammonium) showed a remarkably high cell labeling and internalization efficiency without accompanying significant cytotoxicity. The properties would facilitate not only cell tracking, but also gene delivery applications [5,28]. We believe that the ring-opening method is useful and promising, and will open a new opportunity of nanoparticles for biomedical applications.

## Supplementary Materials

The following are available online at http://www.mdpi.com/1996-1944/11/8/1479/s1, Figure S1: Dispersion states of FND samples; Figure S2: Stability test of FND-HPGTMA; Figure S3: Preparation of FND-PEI; Figure S4: Cell viability testing of MCF7 cells treated with FND-bare and FND-HPGTMA for 24 h using CCK-8. Figure S5: Bright field image, confocal fluorescence images (1 μm/step), and image of bright field and z-stacked fluorescence merged images of HeLa cells treated with (a) 10 μg/mL and (b) 100 μg/mL of FND-HPGTMA.; Figure S6: Single-sliced bright field and fluorescence merged images (FNDs in red; nuclei in blue) and z-stacked FNDs fluorescence images of cells from bottom to top in three dimensions (vertical thickness of 0.5 μm/step). Cells were treated with FND samples at 100 μg/mL for 1 h.
