# Supplementary material for: Single-Step Metal-Free Grafting of Cationic Polymer Brushes on Fluorescent Nanodiamonds
Source: Materials (Basel). 2018 Aug 20;11(8):1479. doi: 10.3390/ma11081479 (PMC6119955; doi:10.3390/ma11081479)
Supplement: Supplementary file 1 [file materials-11-01479-s001.pdf]

materials

Supplementary Materials

# Single-Step Metal-Free Grafting of Cationic Polymer Brushes on Fluorescent Nanodiamonds

**Shingo Sotoma *, Feng-Jen Hsieh and Huan-Cheng Chang ***

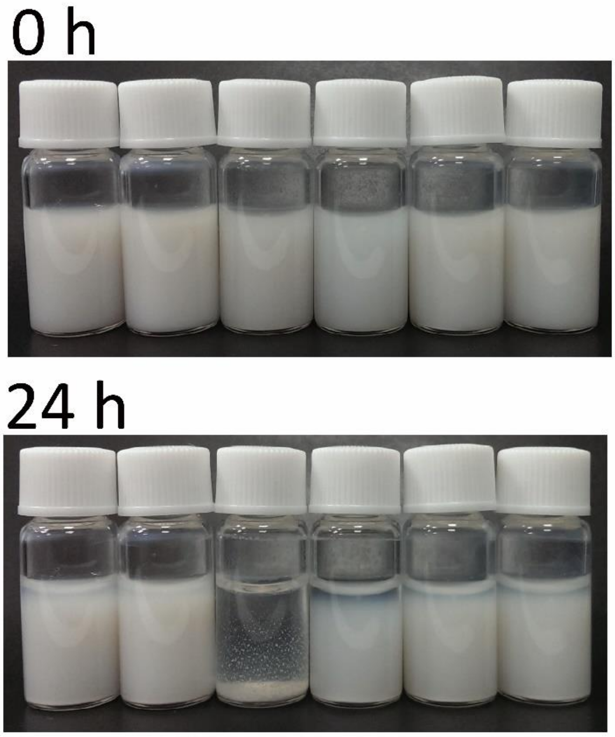


**Figure S1.** Dispersion states of FND samples. FND-bare, FND-HPG, FND-HPGTMA (25%, 50%, and 75%), and FND-PEGTMA in DDW before and after 24 h are shown (from left to right). FND-HPGTMA (25%) precipitated after 24 h presumably because of the lower surface potential.

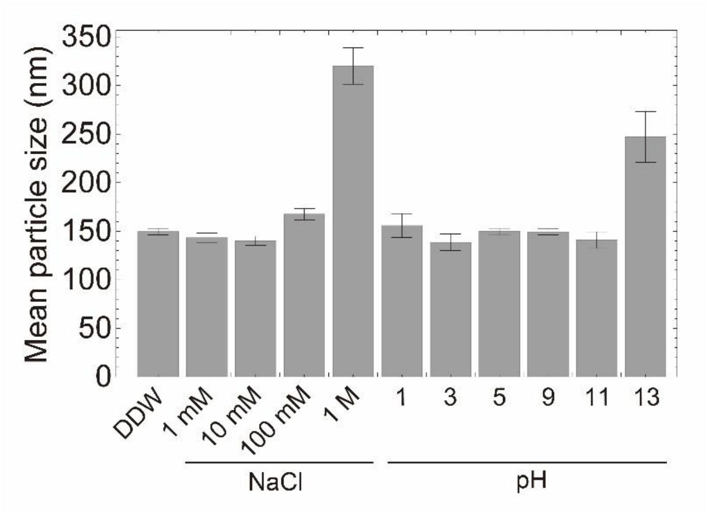


**Figure S2.** Stability test of FND-HPGTMA. Mean particle sizes of FND-HPGTMA in various NaCl concentration and pH aqueous solutions. The data were measured by Zetasizer Nano ZSP, Malvern. Values are means ± standard deviations of three measurements.

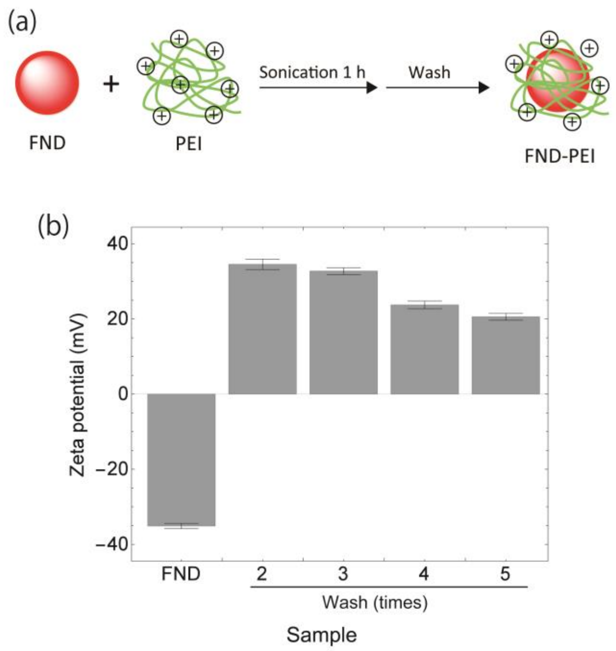


**Figure S3.** Preparation of FND-PEI. (a) Schematic of FND-PEI and (b) Zeta potential values of FND and FND-PEI with various times of washes with DDW. The zeta potential values of FND-PEI decrease with increasing times of wash. In the experiment, we used the sample after second wash. Values are means ± standard deviations of three measurements.

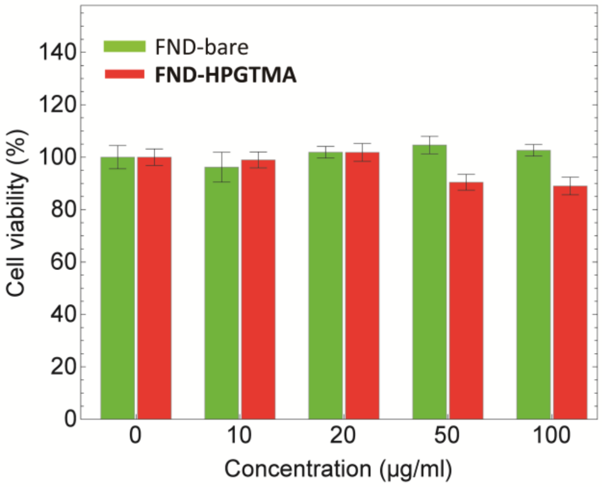


**Figure S4.** Cell viability testing of MCF7 cells treated with FND-bare and FND-HPGTMA for 24 hours using CCK-8. Values are means ± standard deviations of three measurements. More than 90% of the cells were alive after the treatment at 100 μg/mL.

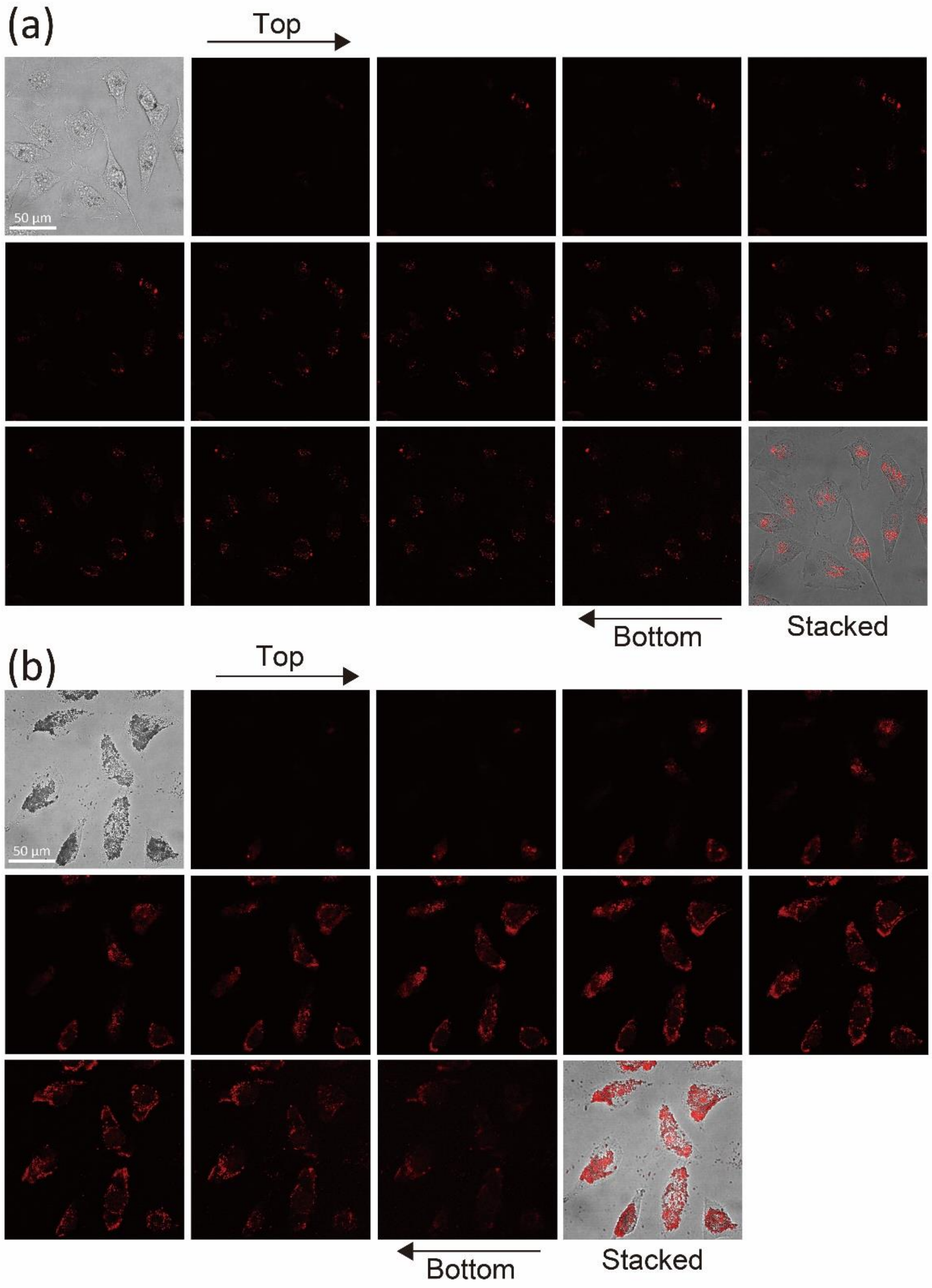


**Figure S5.** Bright field image, confocal fluorescence images (1 μm/step), and image of bright field and z-stacked fluorescence merged images of HeLa cells treated with (a) 10 μg/mL and (b) 100 μg/mL of FND-HPGTMA.

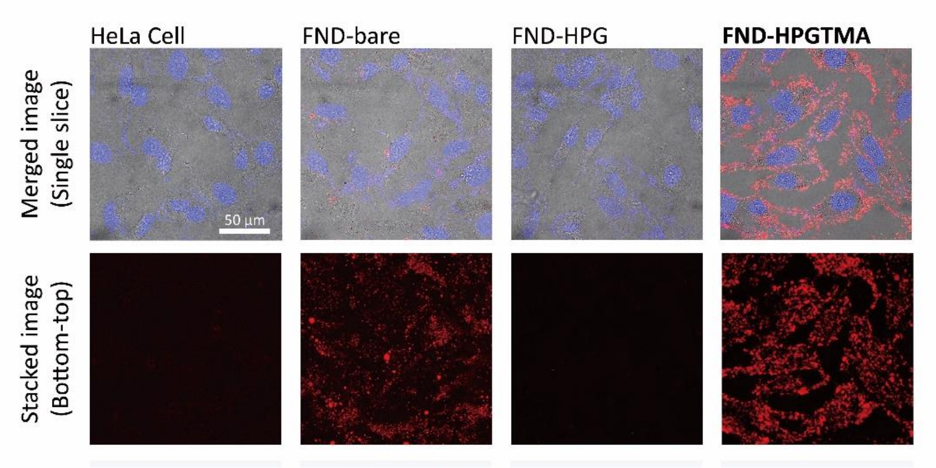


**Figure S6.** Single-sliced bright field and fluorescence merged images (FNDs in red; nuclei in blue) and z-stacked FNDs fluorescence images of cells from bottom to top in three dimensions (vertical thickness of 0.5 μm/step). Cells were treated with FND samples at 100 μg/mL for 1 h.

© 2018 by the authors. Submitted for possible open access publication under the terms and conditions of the Creative Commons Attribution (CC BY) license (http://creativecommons.org/licenses/by/4.0/).
